# Biocontrol and Nanotechnology Strategies for Postharvest Disease Management in Fruits and Vegetables: A Comprehensive Review

**DOI:** 10.3390/foods14162782

**Published:** 2025-08-10

**Authors:** Habiba Lawal, Mohammed Sani Gaddafi, Aasia Muhammed Jamiu, Gerefa Sefu Edo, Opoku Genevieve Fremah, Abdulgaffar Usman El-yakub, Gustav Komla Mahunu, Kaili Wang, Hongyin Zhang, Qiya Yang

**Affiliations:** 1 School of Food and Biological Engineering, Jiangsu University, Zhenjiang 212013, China; habeebalawal1@gmail.com (H.L.); hasiatemjay@gmail.com (A.M.J.); sefgare@gmail.com (G.S.E.); gopoku@htu.edu.gh (O.G.F.); 1000005483@ujs.edu.cn (K.W.); 2 Ministry of Animal Health, Husbandry, and Fisheries, Birnin-Kebbi 860101, Nigeria; gaddafimohammedsani@yahoo.com; 3 Medical Biochemistry Department, Faculty of Basic Medical Sciences, Kaduna State University, Kaduna 800244, Nigeria; abdulgaffarusmane@gmail.com; 4 Department of Food Science & Technology, Faculty of Agriculture, Food and Consumer Sciences, University for Development Studies, Tamale P.O. Box TL1350, Ghana; gmahunu@uds.edu.gh

**Keywords:** biocontrol agents, postharvest losses, fruit, vegetable quality, food security

## Abstract

Postharvest losses in fruits and vegetables, estimated at 20–50% globally, undermine food security and economic stability. Biological control agents (BCAs), including bacteria, yeasts, and fungi, are emerging as eco-friendly alternatives to synthetic fungicides. This review comprehensively analyzes advances in BCAs for postharvest disease control and highlights their mechanisms, impacts on produce quality, and integration into sustainable systems. Additionally, this review delves into the innovative role of nanotechnology-enhanced BCAs (Nano-BCAs), emphasizing nanoencapsulation, improved biofilm formation, targeted delivery, and antimicrobial synergy. While promising, Nano-BCA application requires risk assessment, regulatory clarity, and cost-effective scalability. This synthesis aims to guide future research and application toward sustainable, safe, and efficient postharvest disease management.

## 1. Introduction

Postharvest losses in fruits and vegetables represent one of the most pressing challenges in the global food supply chains, contributing significantly to food insecurity, economic waste, and environmental degradation [1]. It is estimated that between 20 and 50% of all fruits and vegetables produced globally are lost due to decay during the postharvest phase [2]. This challenge is more acute in developing countries, where inadequate infrastructure, limited access to cold storage, and improper handling contribute to the rapid decline in produce quality [3]. Additionally, postharvest losses contribute to greenhouse gas emissions, with decaying organic matter emitting methane, a potent greenhouse gas [4]. As the global population grows and the demand for fresh, high-quality produce increases, addressing postharvest losses becomes increasingly critical to ensuring food security and environmental sustainability [5].

Synthetic chemical fungicides, the commonly applied agents against postharvest disease, have raised several concerns related to human health, environmental pollution, and the development of resistant strains of pathogens [6]. In the search for alternative approaches, biocontrol agents (BCAs) have emerged as a promising, eco-friendly method to manage postharvest diseases, reduce spoilage, and extend the shelf-life of fruits and vegetables [7].

Biocontrol agents are natural organisms (such as bacteria, yeasts, and fungi), natural compounds (such as plant extracts), and other bio-based products (such as biopesticides, biofungicides) that are used to antagonize or control plant pathogens, and their mechanisms of action include competitive exclusion, antibiosis, induced resistance, and parasitism [8]. These agents offer several advantages over synthetic chemical treatments, including the reduction in toxic residues, minimal environmental impact, the ability to target pathogens without harming beneficial organisms, and relatively cost-effective [9]. Furthermore, over the past few decades, BCAs have gained traction in postharvest management due to their compatibility with integrated pest management (IPM) programs and their potential to contribute to sustainable agricultural practices [10].

The use of BCAs in postharvest systems is particularly promising in fruits and vegetables, which are highly perishable and vulnerable to a wide array of microbial pathogens during storage and transportation [11]. Some of the most common pathogens responsible for spoilage include *Botrytis cinerea* (gray mold), *Penicillium expansum* (blue mold), and *Colletotrichum* spp. (anthracnose), which cause significant economic losses across various fruit and vegetable industries [12]. The application of BCAs offers a biological alternative to chemical fungicides, capable of mitigating these pathogens while maintaining the quality and safety of the produce [13].

In addition to their pathogen-suppressing capabilities, BCAs can also enhance the overall quality of fruits and vegetables by delaying ripening, reducing ethylene production, and maintaining moisture content [14]. Such benefits not only extend shelf-life but also improve the sensory attributes and nutritional value of produce, which are key factors in consumer satisfaction and marketability [15]. The growing body of research supporting the efficacy of BCAs in postharvest systems underscores their potential as a cornerstone of sustainable postharvest management strategies [16].

Despite the advantages of BCAs, challenges such as variability in efficacy, sensitivity to environmental conditions, and limited stability during storage and application hinder their widespread adoption [17]. To address these limitations, nanotechnology has emerged as a transformative approach in postharvest disease management [18,19]. Nano-enhanced biocontrol agents (Nano-BCAs) involve the integration of BCAs with nanomaterials such as chitosan, silver nanoparticles (AgNPs), silica-based carriers, and polymeric nanocarriers to enhance stability, improve pathogen targeting, and provide controlled release mechanisms [20]. Nanoencapsulation protects BCAs from desiccation and environmental stress while facilitating their gradual release onto fruit and vegetable surfaces, thereby increasing their persistence and efficacy [21,22]. Additionally, nanocarriers can enhance the adhesion of BCAs to produce surfaces, ensuring uniform colonization and prolonged pathogen suppression [23,24]. Some nanomaterials, such as chitosan and AgNPs, also exhibit intrinsic antimicrobial properties, further augmenting the biocontrol potential of BCAs [25].

**Figure 1:**
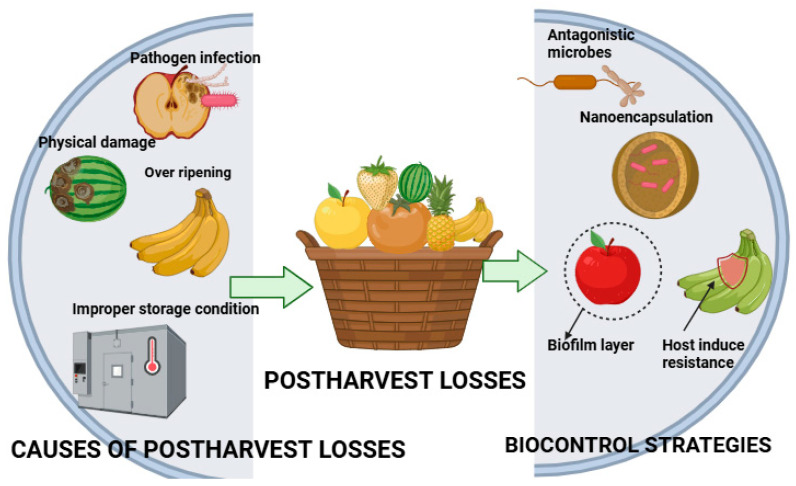
Key causes of postharvest losses and the role of biocontrol strategies created with biorender.com.

The integration of nanotechnology into biocontrol strategies offers multiple benefits, including enhanced stability, improved formulation, reduced application frequency, and greater pathogen suppression efficacy [26]. However, the potential risks associated with nanomaterials, including safety concerns and regulatory challenges, must be critically evaluated to ensure their responsible application in food systems [27]. Future research should focus on optimizing Nano-BCA formulations, assessing their environmental impact, and developing scalable technologies for commercial implementation [28]. Several reviews have explored the biological control of postharvest diseases [29,30,31], yet few have holistically analyzed the synergy between BCAs and nanotechnology. This review aims to fill this critical gap by evaluating the application of Nano-BCAs and their integration into sustainable systems. Figure 1 illustrates the key causes of postharvest losses and the role of biocontrol strategies.

## 2. Postharvest Challenges in Fruits and Vegetables

Fruits and vegetables are essential components of human diets, providing vital nutrients, vitamins, and minerals [32]. However, their highly perishable nature poses significant postharvest challenges that threaten food security, economic stability, and environmental sustainability [33]. These challenges arise from biological, environmental, and infrastructural factors that collectively contribute to substantial losses during storage, transport, and marketing [34]. Postharvest losses of fruits and vegetables are estimated to account for 30–50% of global production, with higher losses recorded in developing countries due to inadequate preservation technologies and infrastructure [35].

One of the major challenges is physiological and microbial spoilage, which affects the quality and safety of fresh produce [36]. After harvest, fruits and vegetables remain metabolically active, undergoing respiration, ethylene production, and senescence [31]. These processes lead to the degradation of texture, color, flavor, and nutritional content [37]. High moisture content and nutrient-rich composition make fruits and vegetables highly susceptible to microbial infections caused by fungi, bacteria, and yeasts, which exacerbate spoilage and pose serious food safety risks [38]. Common pathogens such as *Botrytis cinerea*, *Penicillium* spp., and *Alternaria* spp. are responsible for significant postharvest diseases, reducing the marketability and shelf life of fresh produce [39].

Environmental factors also contribute to postharvest challenges. Improper handling, exposure to high temperatures, and fluctuating humidity during storage and transport accelerate water loss, wilting, and decay [40]. These effects are particularly pronounced in regions with limited access to cold storage facilities or controlled-atmosphere storage systems [31]. Additionally, mechanical injuries sustained during harvesting, packing, and transport create entry points for pathogens, further compromising produce quality [41].

The economic impact of postharvest losses is profound, affecting farmers, distributors, and consumers alike [42]. Smallholder farmers in developing regions are particularly vulnerable, as they often lack access to advanced postharvest technologies, resulting in reduced incomes and increased food insecurity [43]. For consumers, postharvest losses lead to higher prices and reduced availability of fresh produce, exacerbating nutritional deficiencies in vulnerable populations [44].

Environmental sustainability is also undermined by postharvest losses. Spoiled produce contributes to food waste, which has significant environmental repercussions, including the loss of resources used in production, such as water, land, and energy, as well as increased greenhouse gas emissions from decomposing organic matter [45]. Addressing postharvest challenges is, therefore, essential for achieving global sustainability goals, including reducing food waste and ensuring efficient resource utilization [46].

Efforts to mitigate postharvest losses require a multifaceted approach. Technological advancements in postharvest handling, storage, and transportation are critical to preserving quality and extending shelf life [47,48]. For instance, the use of controlled atmospheres, refrigeration, and emerging technologies such as edible coatings and biocontrol agents can significantly reduce spoilage [49]. Public and private investments in cold chain infrastructure, particularly in developing regions, can also minimize losses during transport and storage [50].

Education and capacity-building programs are equally important in empowering farmers and stakeholders to adopt best practices in postharvest management [51]. Integrating traditional knowledge with modern technologies can create context-specific solutions that address local challenges [52]. Policy interventions to promote access to affordable postharvest technologies and incentivize sustainable practices are also vital [53].

## 3. Mechanism of Biocontrol in Postharvest Management

Biocontrol agents have gained increasing recognition as an eco-friendly, sustainable alternative to synthetic fungicides in the management of postharvest diseases of fruits and vegetables [54]. The biocontrol of postharvest pathogens relies on the interaction between the biocontrol organism and the target pathogen, utilizing multiple modes of action to inhibit pathogen growth and spread [55]. The success of BCAs in postharvest systems stems from their ability to target pathogens through various mechanisms, including competition for nutrients and space, parasitism, antibiosis, and induction of host resistance [56]. These mechanisms, either alone or in combination, contribute to the suppression of spoilage organisms, prolonging the shelf-life and maintaining the quality of produce [17,57].

**Figure 2:**
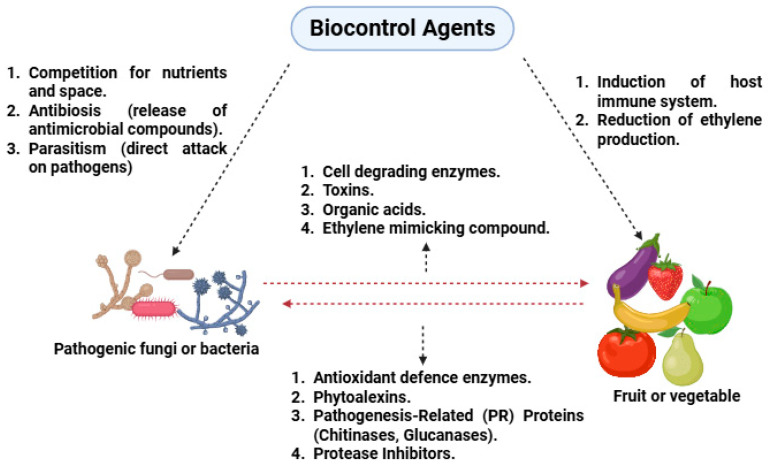
Mechanism of action of biocontrol agents on fruits and vegetables created with biorender.com.

Despite their promise, BCAs face limitations such as sensitivity to storage conditions, inconsistent efficacy under fluctuating temperatures, and challenges in colonization on fruit surfaces. These limitations constrain commercial adoption and require formulation improvements. Figure 2 below summarizes these mechanisms, including antibiosis, competition, and induced resistance.

### 3.1. Competition for Nutrients and Space

One of the primary mechanisms by which biocontrol agents operate is through competition for essential nutrients and ecological niches that pathogens require for their growth and development [58]. Many postharvest pathogens, such as *Botrytis cinerea*, *Penicillium expansum*, and *Colletotrichum* spp., require available sugars, amino acids, and other nutrients to establish infection and produce decay in fruits and vegetables [31,59]. BCAs can outcompete these pathogens by rapidly colonizing the surface of the produce or wound sites, thereby denying the pathogens access to nutrients [60].

For instance, yeasts such as *Candida oleophila* and *Pichia guilliermondii* have been shown to effectively colonize the surfaces of fruits, forming a protective biofilm that limits the availability of resources to fungal pathogens [61]. These yeasts compete for sugars and other nutrients on the fruit surface, reducing the capacity of pathogens like *Penicillium expansum* to initiate infection [62]. This competitive exclusion prevents the establishment of pathogen colonies and helps reduce the incidence of postharvest decay [63].

Similarly, bacteria such as *Pseudomonas fluorescens* and *Bacillus subtilis* are known to colonize wounds on fruits and vegetables, where they can effectively outcompete pathogens for limited resources [64]. This competition-based mechanism is particularly effective in environments with low nutrient availability, where pathogens are more vulnerable to nutrient limitation [65]. By depriving pathogens of the nutrients necessary for their survival, BCAs can significantly reduce the incidence of postharvest infections and spoilage [11].

### 3.2. Parasitism and Hyper Parasitism

Parasitism, or the direct attack of one organism by another, is another key mechanism employed by certain biocontrol agents to suppress postharvest pathogens [66]. In this context, parasitism refers to the ability of a BCA to attach to, penetrate, and kill the pathogen, often through the secretion of lytic enzymes that degrade the pathogen’s cell walls [67].

Fungal BCAs, such as *Trichoderma harzianum* and *Gliocladium catenulatum*, are well-known for their mycoparasitic abilities [68,69]. These fungi produce enzymes such as chitinases, glucanases, and proteases that degrade the cell walls of fungal pathogens, leading to pathogen lysis and death [64]. For example, *Trichoderma harzianum* has been extensively studied for its ability to parasitize postharvest pathogens like *Botrytis cinerea* and *Rhizopus stolonifer*, both of which cause significant spoilage in fruits and vegetables [70]. Upon contact with the pathogen, Trichoderma attaches to its hyphae, secretes cell wall-degrading enzymes, and penetrates the pathogen’s mycelium, resulting in its destruction [71].

In addition to fungal BCAs, bacterial biocontrol agents can also exhibit parasitic behaviour [72,73]. For instance, *Bacillus subtilis* has been reported to produce enzymes that lyse fungal hyphae, disrupting the pathogen’s structure and preventing infection [74]. The hyperparasitic activity of BCAs not only directly reduces pathogen populations but also limits their ability to spread and cause further damage in postharvest environments [59].

### 3.3. Antibiosis

Antibiosis refers to the production of secondary metabolites, such as antibiotics, volatile organic compounds (VOCs), or other toxic substances, by biocontrol agents that inhibit or kill pathogenic organisms [31,75]. This mechanism is particularly effective in preventing the growth and reproduction of pathogens on the surface of fruits and vegetables, thereby reducing spoilage and decay [76].

Many BCAs, including bacteria from the genera Bacillus and Pseudomonas, are known to produce a wide range of antimicrobial compounds [77]. Bacillus subtilis, for example, produces lipopeptides such as iturins and fengycins, which disrupt the cell membranes of fungal pathogens, causing leakage of cellular contents and eventual cell death [78]. These lipopeptides are effective against a broad spectrum of postharvest pathogens, including *Penicillium expansum* and *Aspergillus* spp., which cause significant spoilage in stored fruits like apples and pears [61,70].

Similarly, *Pseudomonas fluorescens* produces phenazine compounds that have strong antifungal properties, inhibiting the growth of pathogens such as *Botrytis cinerea* and Rhizoctonia solani [79]. The production of VOCs by BCAs also plays a role in the inhibition of pathogen growth [64]. Volatile compounds like hydrogen cyanide, 2,4-diacetylphloroglucinol, and pyoluteorin can diffuse through the air and inhibit the growth of fungal spores and hyphae, making them particularly useful in controlling postharvest diseases in storage environments [80].

The effectiveness of antibiosis as a biocontrol mechanism depends on the concentration of the antimicrobial compounds produced, the sensitivity of the target pathogen, and environmental conditions such as temperature and humidity [81]. However, the ability of BCAs to produce a diverse range of antimicrobial substances makes antibiosis a versatile and potent strategy for managing postharvest diseases [65].

### 3.4. Induction of Host Resistance

Another mechanism by which biocontrol agents contribute to the suppression of postharvest pathogens is through the induction of host resistance, also known as systemic resistance [82]. In this process, the application of BCAs triggers the plant’s natural defense mechanisms, enhancing the ability of fruits and vegetables to resist infection by pathogens [78].

BCAs such as *Trichoderma harzianum* and *Pseudomonas fluorescens* are known to induce systemic resistance in plants by activating signaling pathways associated with plant defense, including the salicylic acid, jasmonic acid, and ethylene pathways [5]. This induction of resistance results in the accumulation of defensive compounds such as pathogenesis-related (PR) proteins, phenolics, and phytoalexins, which inhibit the growth of pathogens and prevent the establishment of infections [31]. For instance, the application of Trichoderma species to harvested fruits has been shown to activate the production of PR proteins, which enhance the fruit’s resistance to *Botrytis cinerea* and other postharvest pathogens [83].

The induction of systemic resistance offers a valuable advantage in postharvest management because it enhances the fruit or vegetable’s inherent ability to fight off pathogens without the need for direct intervention [84]. This mechanism is particularly useful in combination with other biocontrol strategies, providing long-lasting protection against spoilage organisms throughout the storage and transportation period [63,85].

### 3.5. Biofilm Formation and Surface Colonization

In addition to direct antagonism, some biocontrol agents employ biofilm formation as a protective mechanism against pathogens [86]. Biofilms are structured communities of microbial cells enclosed in a self-produced extracellular matrix that adheres to surfaces [40]. By forming biofilms on the surface of fruits and vegetables, BCAs can create physical barriers that protect against the attachment and colonization of pathogenic organisms [87].

Yeasts, such as *Candida saitoana* and *Debaryomyces hansenii*, have been shown to form biofilms on the surface of harvested fruits, effectively preventing the establishment of pathogens such as Penicillium and Colletotrichum species [88]. These biofilms not only create a competitive environment by occupying space and resources but also provide a protective layer that prevents the penetration of pathogens into the host tissue [89]. Biofilm-forming BCAs are particularly advantageous in postharvest systems, as they provide continuous protection throughout storage, even under fluctuating environmental conditions [90].

The ability of BCAs to form stable biofilms on fruit surfaces also enhances their persistence and efficacy over time [91]. This long-term colonization is crucial for providing consistent protection against postharvest pathogens, particularly in extended storage periods or during long-distance transportation [92].

## 4. The Role of Biocontrol Agents in Shelf-Life Extension of Fruits and Vegetables

Extending the shelf-life of fruits and vegetables is a major challenge in postharvest management [93]. These highly perishable commodities are susceptible to rapid quality deterioration due to factors such as microbial decay, physiological breakdown, and biochemical changes [94]. Postharvest diseases caused by fungal pathogens such as *Botrytis cinerea*, *Penicillium expansum*, and *Rhizopus stolonifer* are among the primary causes of postharvest losses, leading to significant reductions in shelf-life [36]. To address these challenges, BCAs have emerged as an effective and sustainable alternative to synthetic chemical treatments [95]. Ethylene is a key plant hormone that influences fruit ripening; certain BCAs have been shown to interfere with ethylene biosynthesis, delaying senescence [22].

### 4.1. Reduction in Postharvest Decay Through Pathogen Suppression

The most direct role of BCAs in extending shelf-life is their ability to control postharvest diseases by suppressing the growth and activity of pathogens that cause decay [96]. Fruits and vegetables often provide a favorable environment for the proliferation of spoilage organisms, particularly in storage conditions with high humidity and temperature fluctuations [29]. BCAs, such as antagonistic yeasts, bacteria, and fungi, can inhibit the growth of these pathogens through mechanisms such as competition for nutrients, production of antimicrobial compounds, and parasitism, which ultimately lead to reduced disease incidence [97].

For instance, the yeast *Candida oleophila*, a well-studied biocontrol agent, has been shown to effectively control *Penicillium expansum* and *Botrytis cinerea* in apples and pears by colonizing wound sites on the fruit surface and outcompeting these pathogens for available resources [98]. This suppression of decay-causing pathogens significantly prolongs the storage life of treated fruits [46]. Similarly, bacterial BCAs such as *Pseudomonas syringae* and *Bacillus subtilis* have demonstrated effectiveness in controlling fungal pathogens that cause postharvest rot in strawberries, tomatoes, and other produce, thereby extending their shelf-life [15].

BCAs’ ability to limit pathogen colonization is especially important in extending the marketability of fresh produce, where even minor fungal infections can result in rapid spoilage and quality loss [99]. By maintaining pathogen populations below critical thresholds, BCAs help ensure that fruits and vegetables remain fresh for longer periods during storage, distribution, and retail display [27].

### 4.2. Maintenance of Postharvest Quality

Shelf-life extension is not solely determined by the absence of visible spoilage, but also by the preservation of the sensory and nutritional qualities of the produce [100]. Biocontrol agents play a crucial role in maintaining the postharvest quality of fruits and vegetables by reducing biochemical changes associated with senescence and spoilage [77].

Biocontrol agents can reduce the ethylene production rate, a hormone responsible for ripening and senescence in climacteric fruits [101]. For example, studies have shown that certain BCAs, such as *Trichoderma harzianum* and *Bacillus amyloliquefaciens*, can influence the ethylene biosynthesis pathway in fruits like bananas and tomatoes, resulting in delayed ripening and extended shelf-life [102]. By delaying the ripening process, these BCAs help maintain the firmness, color, and texture of fruits, which are key quality attributes consumers consider during purchasing decisions [101].

In addition, BCAs may influence the antioxidant systems within fruits and vegetables, reducing oxidative stress and delaying the onset of senescence-related changes [103]. For example, the yeast *Debaryomyces hansenii* has been reported to enhance the antioxidant defense mechanisms in citrus fruits, reducing oxidative damage and slowing the senescence process [52]. This leads to the retention of freshness, flavor, and nutritional quality, thereby extending the shelf-life of treated produce [104].

### 4.3. Biofilm Formation and Wound Healing

Biocontrol agents can also promote wound healing in fruits and vegetables, thereby reducing the risk of microbial infections and extending the shelf-life of wounded or damaged produce [29]. Many postharvest pathogens gain entry through wounds or natural openings on the fruit surface, such as lenticels or stem scars. BCAs can colonize these wound sites, forming protective biofilms that prevent pathogen colonization and subsequent decay [105].

For example, the yeast *Candida saitoana* has been shown to form biofilms on the surface of citrus fruits, effectively preventing the penetration of pathogens like *Penicillium digitatum*, which causes green mold in citrus [27]. These biofilms act as a physical barrier that reduces the likelihood of infection and decay, ultimately prolonging the shelf-life of the fruit [106]. The biofilm also serves as a habitat for the BCA to continue its antagonistic activity, creating a dynamic protection system against pathogens over time [107].

**Figure 3:**
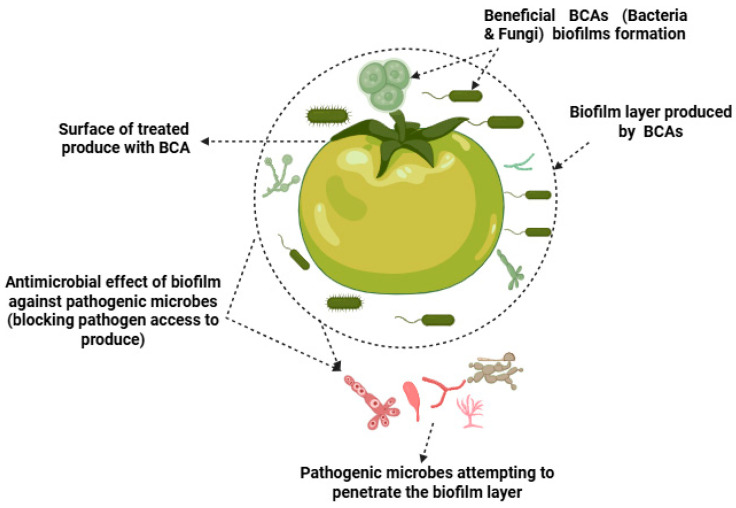
Illustration of biofilm protection of surface of treated produce with BCAs (how BCAs form a biofilm, inhibit pathogen colonization, and protect produce postharvest) created with biorender.com.

Moreover, BCAs may stimulate wound healing by inducing the production of callose, phenolics, and other defensive compounds in the fruit, which reinforce the cell walls and prevent pathogen ingress [108]. This enhanced wound healing capability is particularly valuable for extending the shelf-life of produce that is more susceptible to mechanical damage during harvesting and handling [34,102]. As illustrated in Figure 3, biofilm formation contributes to shelf-life extension by blocking pathogen ingress.

### 4.4. Environmental Suitability and Consistency

One of the advantages of biocontrol agents is their ability to function effectively across a range of environmental conditions, particularly in storage environments where fruits and vegetables are held for extended periods [109]. Unlike synthetic fungicides, which may lose efficacy under certain temperature or humidity conditions, many BCAs are well-suited to operate in both cold and ambient storage environments [110].

For example, *Aureobasidium pullulans*, a widely used yeast-based BCA, has shown robust activity in reducing decay caused by *Penicillium expansum* in apples stored at low temperatures, such as those used in commercial cold storage facilities [111]. Its ability to function at low temperatures is a key factor in maintaining the quality and extending the shelf-life of produce during long-term storage [61].

BCAs can also be integrated into modified atmosphere packaging (MAP) and other postharvest technologies to further extend the shelf-life of produce [112]. Studies have demonstrated that the combination of BCAs with MAP, which modifies the oxygen and carbon dioxide levels within the packaging to slow respiration and microbial growth, can synergistically improve shelf-life extension in fruits like strawberries, grapes, and bell peppers [113].

### 4.5. Safety and Consumer Acceptance

An additional benefit of using BCAs for shelf-life extension is the enhanced consumer acceptance of biocontrol-treated produce [23]. As consumers become increasingly aware of the potential health risks associated with chemical fungicides and the environmental concerns of pesticide overuse, there is a growing demand for organic and residue-free fruits and vegetables [114]. BCAs, being naturally occurring organisms, are perceived as safer and more environmentally friendly alternatives, making them more acceptable to health-conscious consumers [115].

The reduced reliance on synthetic chemicals also aligns with the global movement toward sustainable agricultural practices and organic farming systems [95]. In many countries, the use of BCAs is approved for organic production, allowing growers to meet the standards of organic certification programs while effectively managing postharvest diseases and extending shelf-life [77].

### 4.6. Case Studies on Major Fruits and Vegetables

#### 4.6.1. Apples and Pears

Postharvest decay in apples and pears, largely caused by pathogens like *Penicillium expansum* and *Botrytis cinerea*, leads to significant losses during storage [116]. Synthetic fungicides have traditionally been used to manage these diseases, but BCAs have shown promising results [80]. For instance, Candida sake and *Aureobasidium pullulans* are two yeast-based BCAs that have demonstrated significant efficacy in controlling blue mold (*Penicillium expansum*) and gray mold (*Botrytis cinerea*) in apples and pears [101]. These yeasts colonize wound sites on the fruit, outcompeting pathogens for nutrients and space, and reducing postharvest decay by 60–80% under controlled conditions [117].

#### 4.6.2. Citrus Fruits

Green mold caused by *Penicillium digitatum* is a major postharvest disease in citrus fruits. The yeast *Candida saitoana* has been successfully used to control this pathogen, reducing decay by forming biofilms on citrus fruit surfaces that act as a physical barrier against pathogen colonization [118]. Additionally, the BCA *Pichia guilliermondii* has been shown to suppress green mold in oranges through nutrient competition and the production of antifungal volatile compounds [119]. These biocontrol strategies have significantly reduced green mold incidence in both laboratory and commercial trials, providing a safer alternative to conventional fungicides like thiabendazole [65].

#### 4.6.3. Tomatoes

Tomatoes are highly perishable and prone to postharvest diseases such as *Rhizopus stolonifer* (soft rot) and Alternaria alternata (black mold) [25]. BCAs like *Bacillus subtilis* and *Trichoderma harzianum* have been extensively studied for their ability to control these pathogens in tomatoes [120]. For example, *Bacillus subtilis* produces lipopeptides, which disrupt pathogen membranes, and has been found to reduce soft rot and black mold in tomatoes by 50–70% [64]. In comparison, synthetic fungicides often lead to residue concerns and resistance development in pathogens, making BCAs a viable, safer alternative [94].

#### 4.6.4. Strawberries

Strawberries are vulnerable to *Botrytis cinerea* (gray mold), one of the most destructive postharvest pathogens [121]. The application of Pichia anomala and Candida sake has proven effective in controlling gray mold in strawberries, reducing decay by up to 75% [122]. These BCAs work by competing for space and nutrients on the fruit surface, inhibiting pathogen growth. Additionally, combining BCAs with modified atmosphere packaging (MAP) has been found to further extend the shelf-life of strawberries during cold storage [83].

#### 4.6.5. Leafy Greens

Leafy greens, such as spinach and lettuce, are highly susceptible to *Pseudomonas fluorescens* and *Erwinia carotovora*, which cause soft rot [123]. BCAs like *Bacillus amyloliquefaciens* have demonstrated efficacy in controlling these pathogens by producing antifungal compounds that inhibit pathogen growth [124]. Studies show that applying Bacillus strains can reduce postharvest rot in leafy greens by 40–60%, extending their shelf-life [59].

### 4.7. Impact of Biocontrol Agents on Postharvest Physiology

Biocontrol agents not only reduce decay but also positively influence the postharvest physiology of fruits and vegetables, contributing to extended shelf-life and maintained quality [125]. Their influence extends to ripening processes, ethylene production, respiration rates, and oxidative stress [126].

#### 4.7.1. Delayed Ripening and Reduced Ethylene Production

Ethylene, a plant hormone, accelerates ripening and senescence in climacteric fruits such as bananas, tomatoes, and apples [127]. BCAs, particularly those from the Trichoderma genus, have been shown to reduce ethylene production by interfering with the ethylene biosynthesis pathway [83]. In bananas, the application of *Trichoderma harzianum* delayed ripening by inhibiting the activity of the enzyme ACC oxidase, which is involved in ethylene production. This resulted in extended shelf-life by up to five days compared to untreated controls [128].

#### 4.7.2. Reduction in Respiration Rate

The respiration rate of fruits and vegetables is closely linked to their postharvest longevity. A high respiration rate accelerates metabolic processes, leading to faster deterioration [129]. BCAs like *Pichia guilliermondii* and *Debaryomyces hansenii* have been reported to reduce the respiration rate in apples and citrus fruits by enhancing the antioxidant defense systems of the host [130]. This reduction in respiration rate slows down senescence and extends the postharvest life of the treated produce [131].

#### 4.7.3. Reduction in Oxidative Stress

Oxidative stress is a major contributor to the deterioration of postharvest produce, leading to quality loss and spoilage [132]. Certain BCAs, such as *Candida oleophila* and Pichia membranifaciens, have been shown to enhance the activity of antioxidant enzymes like superoxide dismutase (SOD) and catalase in fruits, thereby reducing oxidative damage during storage [125]. This results in extended shelf-life and improved retention of sensory and nutritional qualities [5].

## 5. Biocontrol Agents and Food Safety

**Figure 4:**
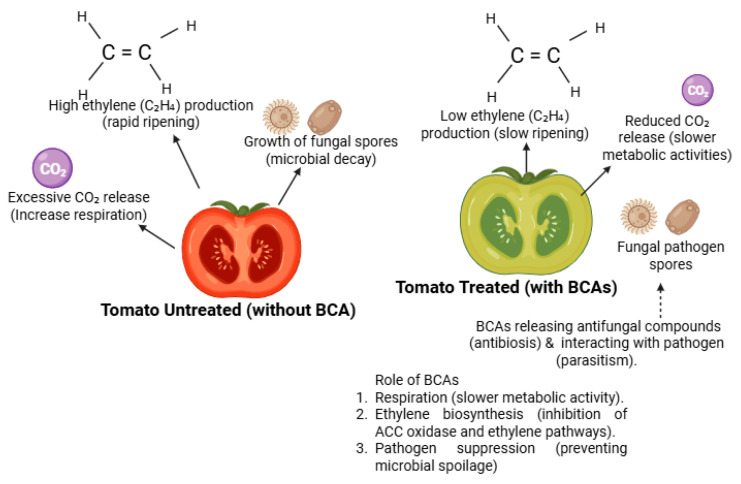
Role of biocontrol agents on postharvest physiology of tomato created with biorender.com.

Food safety is a paramount concern in modern agriculture, particularly in the context of postharvest management of fruits and vegetables [133]. With increasing consumer demand for high-quality, residue-free produce, BCAs have gained attention as an environmentally friendly and safe alternative to synthetic chemicals [58,134]. BCAs provide effective control against postharvest pathogens while offering significant food safety advantages, particularly in terms of microbial safety and the absence of chemical residues [35]. This section explores these critical aspects of BCAs in detail, emphasizing their microbial safety and residue-free nature [135]. Figure 4, higlights the role BCAs play on postharvest physiology of tomaioes.

### 5.1. Microbial Safety of Biocontrol Agents

The use of living microorganisms as BCAs in agriculture raises concerns about the potential introduction of pathogenic microbes into the food supply [136]. However, extensive research has demonstrated that the majority of BCAs used in postharvest management are inherently safe and do not pose significant risks to human health [93]. Biocontrol microorganisms are carefully selected based on their non-pathogenic nature, and they undergo rigorous testing to ensure their safety for both consumers and the environment [137].

#### 5.1.1. Selection and Screening of Non-Pathogenic BCAs

Biocontrol agents are typically selected from naturally occurring microorganisms that exist in the environment, including bacteria, yeasts, and fungi. During the screening process, the potential for pathogenicity, allergenicity, and toxicity is thoroughly evaluated [138]. Only strains that are non-pathogenic and do not produce harmful toxins or allergens are considered suitable for biocontrol applications [139].

For example, *Candida oleophila* and *Pichia guilliermondii* are two yeast species widely used as BCAs in the postharvest management of fruits [125]. These yeasts have been extensively studied and shown to be safe for human consumption [89]. *Candida oleophila*, for instance, has been approved by the U.S. Environmental Protection Agency (EPA) for use on postharvest crops, as it does not pose any adverse effects on human health when applied at recommended levels [83].

#### 5.1.2. Absence of Toxigenic Compounds

Another important aspect of the microbial safety of BCAs is the absence of toxigenic compounds [100]. Unlike synthetic fungicides, which can leave toxic residues on fruits and vegetables, BCAs do not produce harmful secondary metabolites that could compromise food safety [91]. BCAs such as *Aureobasidium pullulans* and *Bacillus subtilis* are known to produce lipopeptides and antifungal enzymes that specifically target pathogens without generating harmful residues [59]. These natural compounds break down quickly and are not harmful to humans, making BCAs a safer option for postharvest disease control [64].

#### 5.1.3. Regulatory Oversight and Compliance

To ensure microbial safety, BCAs are subject to regulatory approval before being introduced to the market [21]. In the United States, the EPA, the Food and Drug Administration (FDA), and the U.S. [101] Department of Agriculture (USDA) collaborate to assess the safety of biocontrol products. In the European Union, the European Food Safety Authority (EFSA) plays a similar role [107]. BCAs must comply with stringent safety standards, including the absence of harmful contaminants, potential antibiotic resistance genes, and adverse effects on non-target organisms. Such regulatory oversight ensures that only safe and effective BCAs are used in food production systems [22].

### 5.2. Residue-Free Benefits of Biocontrol Agents

One of the most significant advantages of BCAs over synthetic chemicals is their residue-free nature [7]. Synthetic fungicides, which are commonly used to control postharvest diseases, often leave chemical residues on fruits and vegetables, raising concerns about food safety, environmental impact, and public health [40]. Biocontrol agents, on the other hand, offer a residue-free solution to postharvest disease management, enhancing both food safety and consumer confidence [70].

#### 5.2.1. Chemical Residue Concerns with Synthetic Fungicides

The use of synthetic fungicides in postharvest treatment has raised significant food safety concerns due to the presence of chemical residues on harvested produce [140]. Residues of fungicides such as thiabendazole, imazalil, and fludioxonil have been detected on a wide range of fruits and vegetables, often exceeding maximum residue limits (MRLs) established by regulatory agencies [141]. These residues pose potential risks to human health, including allergic reactions, endocrine disruption, and long-term carcinogenic effects [136]. Additionally, frequent exposure to pesticide residues through food consumption has contributed to the development of pesticide resistance in pathogens, further complicating disease management [142,143].

#### 5.2.2. BCAs as a Residue-Free Alternative

BCAs present a residue-free alternative to synthetic chemicals, as they are composed of naturally occurring microorganisms that do not leave harmful residues on treated produce [114]. *Pichia guilliermondii* and *Bacillus subtilis* are naturally occurring microbes widely studied for their safety and effectiveness. Both have GRAS (Generally Recognized As Safe) status, approved for postharvest use by regulatory agencies like the EPA [144,145]. For instance, the application of *Pichia guilliermondii* and *Bacillus subtilis* on citrus fruits and tomatoes, respectively, has been shown to effectively control postharvest diseases without leaving detectable residues [146]. Since BCAs are biological, they decompose into harmless byproducts, ensuring that no chemical residues remain on the fruits and vegetables after treatment [16].

The absence of chemical residues also makes BCAs an ideal choice for organic agriculture, where the use of synthetic pesticides is strictly regulated [20]. Organic farming systems prioritize the use of natural and sustainable methods for disease control, and BCAs align perfectly with these principles [147]. Their use in organic postharvest management ensures compliance with organic certification standards while maintaining the quality and safety of the produce [148].

#### 5.2.3. Consumer Preferences and Market Demand

Consumer preferences for residue-free produce have grown significantly in recent years, driven by increased awareness of the potential health risks associated with chemical pesticides [149]. Surveys indicate that consumers are willing to pay a premium for fruits and vegetables that are free from pesticide residues and produced using environmentally friendly practices [138]. The use of BCAs not only enhances food safety but also provides a marketing advantage by meeting consumer demand for clean, safe, and sustainable food products [113,150].

Furthermore, the use of BCAs supports retailers’ efforts to comply with stringent food safety regulations, such as the European Union’s Farm-to-Fork strategy, which aims to reduce the use of chemical pesticides and promote sustainable agricultural practices [151]. Retailers who source produce treated with BCAs can offer consumers high-quality, residue-free products, contributing to increased market competitiveness [152].

## 6. Nanotechnology in Enhancing Biocontrol Efficacy

The integration of nanotechnology into postharvest biocontrol strategies has led to significant advancements in the efficacy, stability, and delivery of BCAs [97]. The major limitations of conventional BCAs include their vulnerability to environmental stress, short shelf-life, poor adhesion to fruit and vegetable surfaces, and limited ability to control pathogens over extended storage periods [153]. Nanotechnology addresses these challenges through four primary mechanisms: nanoencapsulation for stability and controlled release, enhanced adherence and biofilm formation, nanoparticle-mediated antimicrobial activity, and targeted delivery systems for precision application [154] Table 1. These mechanisms contribute to more effective pathogen suppression, prolonged postharvest protection, and reduced dependence on synthetic chemical fungicides [155].

### 6.1. Nanoencapsulation for Stability and Controlled Release

**Figure 5:**
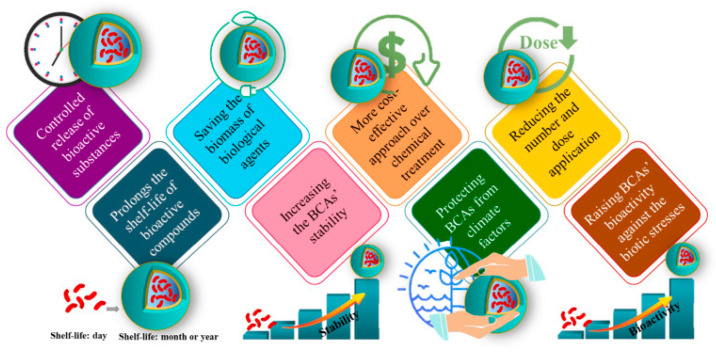
The advantage of encapsulating biological control agents [20].

**Figure 6:**
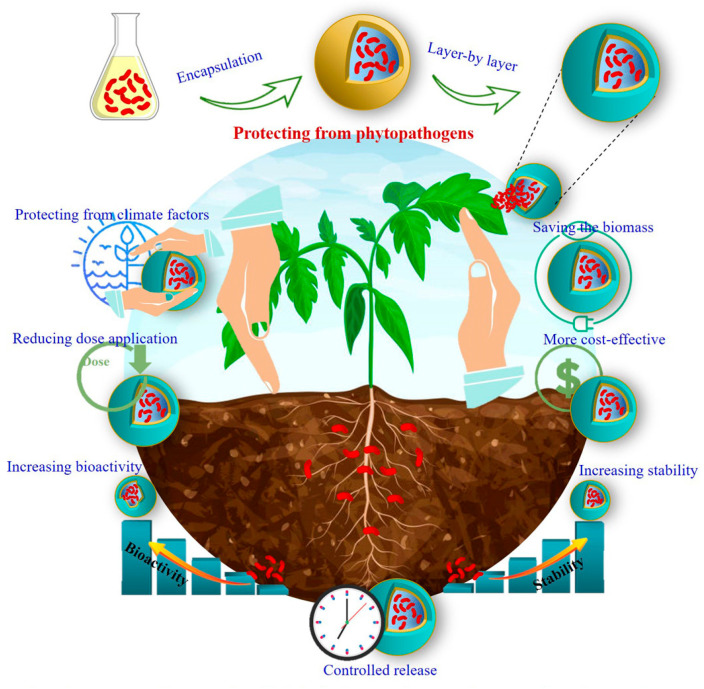
The advantage of encapsulated biological control agents in protecting plant pathogens [20].

Nanoencapsulation significantly enhances the stability and functionality of BCAs by protecting them from environmental stressors such as desiccation, ultraviolet (UV) radiation, and temperature fluctuations [20]. Encapsulating BCAs in nanocarriers, such as chitosan nanoparticles, liposomes, and polymeric nanospheres, extends their viability and ensures a sustained release profile [156]. This controlled release mechanism allows BCAs to remain active for longer durations, thereby improving their effectiveness against postharvest pathogens without requiring frequent reapplication [97]. For instance, encapsulated *Trichoderma harzianum* in chitosan-based nanoparticles demonstrated extended antagonistic activity against *Botrytis cinerea*, a major postharvest pathogen in fruits and vegetables, by modulating the slow release of viable fungal spores over time [157]. Additionally, nanoencapsulation can protect BCAs from degradation during transportation and storage, ensuring their potency at the time of application [155]. The enhanced stability and controlled release of nano-formulated BCAs not only improve their reliability but also reduce application costs and labor, making biocontrol strategies more feasible for large-scale agricultural practices [158]. Figure 5 and Figure 6 demonstrate the protective effect of nanoencapsulation on BCAs against environmental stress

### 6.2. Enhanced Adherence and Biofilm Formation on Fruit Surfaces

One of the fundamental challenges in postharvest biocontrol is ensuring that BCAs effectively colonize fruit and vegetable surfaces, creating a stable microbial community that prevents pathogen invasion [30]. Many conventional BCAs fail to establish a strong attachment due to their hydrophilic nature and weak interactions with the hydrophobic surfaces of fruits and vegetables [65]. Nanotechnology provides a solution by modifying the physicochemical properties of BCAs, thereby enhancing their adhesion and biofilm formation [159].

Nanoparticles such as chitosan, silica, and lipid-based nanocarriers can act as carriers that promote stronger electrostatic and hydrophobic interactions between BCAs and fruit surfaces [160]. Chitosan-based nanoparticles, in particular, have been shown to enhance the attachment of *Pichia guilliermondii* on citrus fruit surfaces, preventing the colonization of *Penicillium digitatum*, the causal agent of green mold [161]. Furthermore, nano-coatings made from biodegradable polymers can facilitate the uniform distribution of BCAs across fruit surfaces, preventing the detachment of biocontrol organisms during postharvest handling and washing [162].

In addition to improving attachment, nanotechnology plays a crucial role in enhancing biofilm formation by BCAs, which further strengthens their ability to suppress pathogens [163]. Biofilms are structured microbial communities that form protective layers on fruit and vegetable surfaces, preventing the establishment of fungal and bacterial pathogens [164]. Nano-formulated BCAs exhibit increased biofilm formation due to their ability to retain moisture and nutrients, which are essential for microbial proliferation [165]. For example, *Bacillus subtilis* encapsulated in a lipid-based nano-coating demonstrated enhanced biofilm formation on strawberry surfaces, significantly reducing the incidence of *Botrytis cinerea* infections during storage [166]. The improved adhesion and biofilm-forming capabilities of nano-enhanced BCAs provide long-term protection against postharvest pathogens, ensuring extended shelf-life and better-quality produce [167].

### 6.3. Nanoparticle-Mediated Antimicrobial Activity

Nanoparticles possess unique antimicrobial properties that can complement the activity of BCAs, creating a synergistic effect that enhances pathogen suppression [168]. Several nano-materials, including AgNPs, ZnO-NPs, CuO-NPs, and carbon-based nanomaterials, exhibit potent antimicrobial activity against a wide range of postharvest pathogens [169]. These nanoparticles function through multiple mechanisms, such as disrupting microbial cell membranes, inhibiting metabolic enzymes, generating reactive oxygen species, and interfering with pathogen signaling pathways [170,171]. For example, AgNPs penetrate microbial cell walls, causing structural disintegration.

One of the most extensively studied antimicrobial nanoparticles is AgNP, which has been shown to exhibit strong antifungal and antibacterial activity when combined with BCAs [172]. Studies have demonstrated that AgNPs loaded with *Candida oleophila* significantly inhibited the growth of *Penicillium expansum* on apples, reducing blue mold incidence by 85% compared to non-nanoformulated BCAs [173]. Similarly, ZnO-NPs have been used in conjunction with *Bacillus amyloliquefaciens* to control *Alternaria alternata* in tomatoes, with enhanced suppression observed due to the dual action of ZnO-mediated pathogen inhibition and BCA-induced competition [174].

In addition to their direct antimicrobial effects, nanoparticles can enhance the production of secondary metabolites, enzymes, and antibiotics by BCAs, further boosting their biocontrol efficacy [168]. For instance, the presence of CuO-NPs in *Pseudomonas fluorescens* formulations has been reported to increase the synthesis of phenazines, which are antimicrobial compounds that inhibit fungal growth [175]. This ability of nanoparticles to enhance the secondary metabolite production of BCAs represents an innovative approach to strengthening biocontrol strategies against postharvest pathogens [176].

### 6.4. Targeted Delivery Systems for Precision Application

Nanotechnology enables targeted and precision-based delivery of BCAs to specific infection sites, reducing waste and ensuring maximum efficacy [177]. Conventional application methods for BCAs, such as spraying or dipping, often lead to uneven distribution and rapid loss due to environmental exposure [178]. Nanocarriers allow for controlled, localized, and site-specific release, ensuring that BCAs reach the most vulnerable areas of the fruit or vegetable surface where pathogen attack is most likely [179].

Smart delivery systems, including pH-responsive, temperature-sensitive, and moisture-activated nanoparticles, can be designed to release BCAs only under favorable conditions for pathogen growth, thereby optimizing their activity [180]. For example, pH-responsive nano-formulations of *Aureobasidium pullulans* have been developed to release BCAs at specific pH thresholds that favor fungal colonization, ensuring that the biocontrol agent is deployed precisely when needed [181]. Similarly, temperature-sensitive nano-delivery systems have been used to encapsulate *Pichia membranifaciens*, ensuring its controlled release at cold storage temperatures while maintaining stability at higher temperatures during transportation [182].

Another emerging area in nanotechnology-driven biocontrol is the use of magnetically guided nanoparticles, which allow for BCAs to be precisely delivered to infected areas using external magnetic fields [183]. This technique has been explored for the targeted control of fungal infections in apples and pears, where magnetized nano-formulations successfully adhered to wound sites, preventing pathogen establishment [184]. The ability to direct and regulate BCA application using nanotechnology ensures higher efficiency, reduced environmental impact, and lower production costs for postharvest disease management [185].

Table 1 provides a detailed overview of commonly used BCAs, their mechanisms, nano-enhancement strategies, and target pathogens across various fruits and vegetables.

**Table 1:** Biocontrol Agents and Nanotechnology-Enhanced Biocontrol Agents for Postharvest Management of Fruits and Vegetables.

Fruit/Vegetable	Biocontrol Agent	Target Pathogen(s)	Mode of Action	Nanotechnology Enhancement	References
**Apple**	*Aureobasidium pullulans*	*Penicillium expansum*, *Botrytis cinerea*	Competitive exclusion, biofilm formation, and nutrient competition	Nano-encapsulation in chitosan nanoparticles for prolonged stability and controlled release	[20,181]
**Banana**	*Candida oleophila*	*Colletotrichum musae*, *Fusarium oxysporum*	Competitive exclusion, quorum sensing interference	Coating with lipid-based nanoparticles for enhanced adhesion and controlled application	[182,183]
**Tomato**	*Trichoderma harzianum*	*Botrytis cinerea*, *Rhizoctonia solani*	Mycoparasitism, enzyme production (chitinase, glucanase), and induction of host resistance	Nano-biofilm technology for improved colonization and pathogen suppression	[184,185,186]
**Grapes**	*Pichia guilliermondii*	*Botrytis cinerea*	Competitive exclusion, biofilm formation, and volatile antifungal compound production	Nano-silver coating enhances pathogen suppression and prevents oxidation	[167,187]
**Citrus (Orange, Lemon)**	*Bacillus subtilis*	*Penicillium digitatum*, *Penicillium italicum*	Antibiosis via lipopeptide production, induction of systemic resistance	Encapsulation in pH-responsive nanoparticles for targeted pathogen inhibition	[188,189]
**Peach**	*Metschnikowia fructicola*	*Monilinia laxa*, *Rhizopus stolonifer*	Nutrient competition, host resistance induction, and volatile organic compound (VOC) production	Chitosan nano-coating prolongs BCA activity and reduces fruit respiration	[190,191]
**Strawberry**	*Pseudomonas fluorescens*	*Botrytis cinerea*, *Alternaria alternata*	Siderophore production, hydrogen cyanide (HCN) antifungal activity, and ISR activation	Nanoemulsion-based formulation improves retention and enhances biocontrol efficiency	[192,193]
**Papaya**	*Debaryomyces hansenii*	*Colletotrichum gloeosporioides*, *Aspergillus* spp.	Osmo-tolerance, biofilm formation, and antimicrobial peptide secretion	Nano-chitosan incorporation enhances biofilm formation and adhesion to fruit surfaces	[194,195]
**Mango**	*Trichoderma viride*	*Colletotrichum gloeosporioides*	Mycoparasitism, competitive exclusion, and secondary metabolite production	Liposome-mediated delivery improves BCA survival and efficacy under varying storage conditions	[196,197]
**Blueberry**	*Bacillus amyloliquefaciens*	*Alternaria alternata*, *Botrytis cinerea*	Antibiosis (iturin, fengycin production), nutrient competition, and biofilm formation	Zinc oxide nanoparticle synergy enhances antimicrobial action and fruit shelf-life	[198,199]
**Avocado**	*Bacillus subtilis*	*Colletotrichum gloeosporioides*	Antibiotic production, biofilm formation, and competition for space	Encapsulation in biodegradable nanogels increases colonization and moisture retention	[200,201]
**Cherry**	*Metschnikowia pulcherrima*	*Botrytis cinerea*, *Rhizopus stolonifer*	Nutrient competition, production of antifungal volatiles, and disruption of pathogen quorum sensing	Nano-coating with essential oil nanoparticles enhances antifungal activity	[202,203]
**Cucumber**	*Gliocladium virens*	*Pythium aphanidermatum*, *Fusarium solani*	Hyperparasitism, nutrient competition, and production of gliotoxin	Biodegradable nano-polysaccharide carriers improve stability and pathogen suppression	[204,205]
**Bell Pepper**	*Pseudomonas chlororaphis*	*Phytophthora capsici*	Induced systemic resistance, siderophore production, and competitive exclusion	Electrospun nanofiber delivery system improves adhesion and persistence	[206,207]
**Pineapple**	*Pichia kluyveri*	*Thielaviopsis paradoxa*, *Ceratocystis paradoxa*	Competitive exclusion, disruption of fungal spore germination, and biofilm formation	Nanoencapsulation using alginate nanoparticles improves pathogen suppression	[208,209]
**Melon**	*Pseudomonas putida*	*Fusarium oxysporum*, *Rhizopus stolonifer*	Induced systemic resistance, production of siderophores, and nutrient competition	Smart polymeric nanoparticles for controlled release and pathogen-specific activation	[210,211]
**Carrot**	*Bacillus pumilus*	*Alternaria* spp., *Penicillium* spp.	Antibiosis through antimicrobial peptides, induced systemic resistance, and competitive exclusion	Nano-lipid formulations extend BCA survival under fluctuating storage conditions	[212,213]
**Cabbage**	*Pseudomonas syringae*	*Sclerotinia sclerotiorum*, *Botrytis cinerea*	Siderophore production, nutrient competition, and biofilm formation	Nano-biosensor-integrated application enables precision biocontrol and reduced spoilage	[214,215]
**Lettuce**	*Bacillus cereus*	*Rhizoctonia solani*, *Botrytis cinerea*	Antibiotic production, competition for nutrients, and induction of plant defense responses	Encapsulation in biodegradable nanoparticles enhances BCA adhesion and shelf-life extension	[56,151]

## 7. Sustainability and Environmental Impacts of Nanotechnology-Enhanced Biocontrol Agents

The integration of BCAs in postharvest management has been widely recognized as a sustainable approach to mitigating food losses, reducing chemical fungicide dependency, and preserving environmental health [186]. However, conventional BCAs face several limitations, including variable efficacy, environmental sensitivity, and inconsistent colonization on fruit and vegetable surfaces [64]. These challenges hinder their large-scale adoption and limit their practical application in commercial postharvest systems [18]. Nanotechnology offers a transformative solution to these constraints by enhancing the stability, precision, and efficacy of BCAs while promoting environmentally sustainable practices [58]. Nanotechnology-enhanced biocontrol agents present a significant advancement toward reducing synthetic agrochemical inputs, minimizing environmental contamination, and improving food safety, aligning with global sustainability goals in agriculture and food production [187].

One of the primary advantages of Nano-BCAs is their potential to reduce the reliance on chemical fungicides, which have been associated with environmental pollution, non-target toxicity, and the emergence of resistant pathogen strains [20,187]. Synthetic fungicides leave chemical residues on food products and contribute to soil and water contamination, posing long-term ecological risks [110]. In contrast, Nano-BCAs, particularly those formulated with biodegradable nanocarriers such as chitosan, alginate, and lipid-based nanoparticles, provide a residue-free alternative that ensures effective pathogen suppression without compromising environmental integrity [188]. Encapsulation of BCAs within nanostructured delivery systems protects them from degradation, enhances their colonization efficiency, and enables controlled release, reducing the need for frequent reapplications [189]. This not only lowers the overall chemical load in agricultural ecosystems but also decreases the risk of fungicide residues accumulating in food supply chains [190].

In addition to their role in reducing chemical inputs, Nano-BCAs contribute to enhanced ecosystem health by mitigating agricultural pollution and promoting microbial biodiversity [191]. Conventional BCA formulations often suffer from low retention on fruit surfaces and susceptibility to wash-off during handling and storage, resulting in environmental dispersal and unintended microbial imbalances [192]. Nanocarriers improve the adhesion and persistence of BCAs, ensuring prolonged antagonistic activity against postharvest pathogens while minimizing off-target effects [193]. The use of biodegradable and food-grade nanomaterials further supports soil and water conservation by preventing the accumulation of persistent synthetic compounds [194]. Furthermore, the ability of nanomaterials to enhance biofilm formation by BCAs contributes to more effective microbial colonization on fruit surfaces, creating a protective barrier that limits pathogen establishment and reduces spoilage [195]. This natural bioprotection mechanism aligns with ecological principles of pest suppression and supports sustainable agricultural practices that reduce dependency on synthetic interventions [196].

Beyond direct environmental benefits, Nano-BCAs also offer significant contributions to climate resilience and carbon footprint reduction in postharvest management [197]. Postharvest losses are a major contributor to greenhouse gas emissions, with food spoilage leading to increased methane production and unnecessary energy consumption in cold storage facilities [198]. The extended shelf life and improved pathogen control provided by Nano-BCAs reduce spoilage rates, decreasing food waste and lowering the carbon footprint associated with food production, storage, and transportation [199]. Additionally, Nano-BCAs can reduce the need for energy-intensive refrigeration and chemical fumigation, both of which are critical but environmentally costly components of postharvest disease management [200]. By maintaining fruit and vegetable quality for longer durations, Nano-BCAs indirectly contribute to more energy-efficient supply chains and sustainable food distribution systems [201].

Despite their promising advantages, the widespread adoption of Nano-BCAs in postharvest disease management is subject to regulatory considerations, cost constraints, and potential ecological concerns [202]. While nanotechnology-based formulations have demonstrated improved efficacy, concerns regarding nanoparticle accumulation, biodegradability, and long-term effects on human health and the environment necessitate comprehensive risk assessments and standardized regulatory frameworks [203]. Ensuring that nanomaterials used in biocontrol applications are non-toxic, environmentally degradable, and compliant with food safety regulations is crucial for their acceptance and commercialization [204]. Moreover, the cost of nano-formulation and large-scale production remains a limiting factor for many agricultural stakeholders, particularly in resource-limited settings [18]. Future research should focus on the development of cost-effective, scalable, and eco-friendly nanomaterial synthesis methods that enhance accessibility while maintaining environmental sustainability [205].

The application of nanotechnology in biocontrol strategies represents a major step toward achieving sustainable postharvest management practices [206]. By integrating nanomaterials with BCAs, it is possible to improve pathogen suppression, extend shelf life, and reduce reliance on chemical treatments, leading to safer food production systems and lower environmental impact [207]. The ability of Nano-BCAs to provide targeted, efficient, and long-lasting disease control supports the transition to climate-smart agricultural practices while addressing critical challenges related to food security and waste reduction [208]. However, realizing the full potential of this technology requires interdisciplinary collaborations among food scientists, microbiologists, nano-technologists, and policy-makers to ensure responsible innovation and regulatory alignment [113]. As global food systems continue to evolve in response to environmental and consumer-driven demands, the role of Nano-BCAs in sustainable postharvest management is expected to expand, offering a viable pathway toward greener, more resilient food supply chains [209].

## 8. Challenges and Limitations of Nanotechnology-Enhanced Biocontrol Agents in Agricultural Practices

Despite the significant potential of Nano-BCAs in postharvest disease management, several challenges and limitations hinder their large-scale application in agricultural practices [20]. These challenges range from formulation stability, cost constraints, and regulatory hurdles to potential environmental and human health concerns. Addressing these limitations is essential to facilitate the widespread adoption of Nano-BCAs in commercial agriculture while ensuring their safety, efficacy, and sustainability [207].

One of the primary challenges in utilizing Nano-BCAs is the complexity of formulation and stability [210]. While nanocarriers enhance the viability, controlled release, and adherence of biocontrol agents, maintaining the stability of these nanoformulations under diverse environmental conditions remains difficult [97]. Factors such as temperature fluctuations, humidity variations, and exposure to ultraviolet (UV) radiation can alter nanoparticle integrity and reduce their effectiveness in delivering viable BCAs [188]. Moreover, interactions between nanoparticles and microbial cells may sometimes lead to altered metabolic activities or reduced viability, impacting the biocontrol potential of the encapsulated agents [211]. Achieving an optimal balance between nanoparticle protection and microbial activity requires further optimization of nanoencapsulation techniques and material selection [152]. Additionally, the scalability of nano-formulated BCAs presents a major bottleneck, as laboratory-scale formulations often fail to maintain efficacy when transitioned to large-scale production [16]. Developing standardized, reproducible, and economically viable nano-formulation processes is necessary to bridge the gap between research and commercial application [212].

The high cost of nanomaterial synthesis and nanoencapsulation processes poses another significant limitation to the widespread adoption of Nano-BCAs in agricultural practices [31]. The production of food-grade, biodegradable nanocarriers, such as chitosan, liposomes, or polymeric nanoparticles, requires advanced manufacturing facilities, specialized equipment, and precise formulation techniques, all of which contribute to increased production costs [59]. This cost factor may limit accessibility, particularly for smallholder farmers and agricultural industries in developing regions where cost-effective solutions are critical for implementation [184]. Additionally, the need for cold-chain storage or specialized handling protocols for certain Nano-BCA formulations further increases operational expenses, making them less competitive compared to conventional synthetic fungicides, which are often cheaper and more readily available [183]. To ensure broader adoption, there is a need for innovative cost-reduction strategies, including the use of agro-industrial byproducts for nanoparticle synthesis, large-scale bioprocess optimization, and government incentives to support the transition from chemical-intensive postharvest treatments to sustainable Nano-BCA-based alternatives [203,204].

Regulatory hurdles and safety concerns represent another major challenge in the commercialization of Nano-BCAs [158]. Despite the growing body of evidence supporting their efficacy and environmental benefits, nanotechnology applications in food systems remain subject to strict regulatory scrutiny due to uncertainties regarding nanoparticle toxicity, persistence, and potential bioaccumulation [213]. Regulatory agencies such as the U.S. Food and Drug Administration (FDA), the European Food Safety Authority (EFSA), and the Codex Alimentarius Commission require rigorous safety evaluations before approving nanotechnology-based products for agricultural and food applications [214]. However, there is currently no globally harmonized framework for assessing the risks and benefits of nano-formulated biocontrol agents, leading to inconsistencies in approval processes across different countries [215]. The lack of standardized protocols for evaluating the safety, environmental impact, and long-term fate of nanoparticles further complicates regulatory compliance and delays market entry for Nano-BCA-based formulations [216]. Addressing these challenges requires coordinated efforts between regulatory bodies, researchers, and industry stakeholders to develop clear guidelines that balance safety assessments with innovation, enabling the responsible use of nanotechnology in postharvest disease management [216].

Potential environmental and human health concerns associated with Nano-BCAs also need to be thoroughly examined [59]. While biodegradable nanocarriers such as chitosan and lipid-based nanoparticles are generally considered safe, the ecotoxicological effects of metal-based nanoparticles, such as AgNPs, ZnO-NPs, and CuO-NPs, remain a subject of concern [217]. These nanoparticles have been shown to exhibit antimicrobial properties that may unintentionally disrupt beneficial microbial communities in soil and water ecosystems [218]. Additionally, the fate and persistence of nanoparticles in food products and the human digestive system require further investigation, as the long-term effects of chronic exposure to nanoparticle residues in food are not yet fully understood [219]. Consumer acceptance of nanotechnology-based agricultural products is another challenge, as public perception of nanotechnology in food systems is often influenced by concerns over potential toxicity, ethical considerations, and a lack of transparent labeling [220]. Public education, risk communication, and robust scientific studies on the safety of Nano-BCAs are essential to addressing these concerns and fostering consumer confidence in nanotechnology-enhanced biocontrol strategies [221].

Technical limitations related to targeted delivery and application methods also present obstacles to the effectiveness of Nano-BCAs [170]. While nanocarriers improve the precision and controlled release of BCAs, ensuring uniform distribution on fruit and vegetable surfaces remains challenging, especially in large-scale postharvest operations [174]. The effectiveness of Nano-BCAs can be influenced by factors such as surface properties of the produce, application methods (e.g., spraying, dipping, or coating), and compatibility with existing postharvest handling practices [178]. Additionally, the development of smart nano-delivery systems, such as pH-responsive or temperature-sensitive nanoparticles, is still in its early stages and requires further refinement before large-scale adoption [222]. Future research should focus on advancing intelligent nanocarrier designs that respond to specific environmental triggers, optimizing application technologies for uniform coverage, and integrating Nano-BCAs with existing postharvest preservation methods to maximize their impact [20].

Despite these challenges, the potential of Nano-BCAs in agricultural practices remains substantial, provided that key limitations are addressed through scientific innovation, regulatory advancements, and strategic industry collaborations [187]. Interdisciplinary research efforts combining nanotechnology, microbiology, food science, and precision agriculture will be crucial in overcoming existing barriers and ensuring the safe, cost-effective, and sustainable implementation of Nano-BCAs [203]. By refining nano-formulation techniques, developing eco-friendly nanomaterials, streamlining regulatory pathways, and improving public awareness, Nano-BCAs can play a pivotal role in reshaping postharvest disease management, reducing global food losses, and contributing to a more sustainable and resilient food system [187,204].

## 9. Future Directions of Nanotechnology-Enhanced Biocontrol Agents in Sustainable Agriculture

The integration of nanotechnology with biocontrol strategies represents a significant advancement in sustainable agriculture, particularly in postharvest disease management [204]. Nanotechnology-enhanced biocontrol agents have demonstrated their potential to improve stability, efficacy, and targeted delivery of beneficial microorganisms, reducing reliance on synthetic fungicides and minimizing environmental contamination [20]. However, to fully realize their potential, future research must address key challenges and explore innovative approaches that enhance their functionality, scalability, and regulatory acceptance [185]. The continued development of Nano-BCAs in sustainable agriculture will require interdisciplinary collaboration, advances in nano-formulation techniques, and improved understanding of their ecological interactions to optimize their performance while ensuring food safety and environmental sustainability [194].

One of the primary areas for future exploration is the development of intelligent and stimuli-responsive nanocarriers that can further enhance the precision and efficiency of Nano-BCAs [223]. While existing nanoencapsulation technologies improve the stability and controlled release of BCAs, more advanced delivery systems are needed to ensure their on-demand release in response to environmental cues such as pH shifts, humidity levels, or pathogen presence [224]. The incorporation of smart nanomaterials, such as temperature-sensitive, enzymatically activated, or pH-responsive nanoparticles, can allow for site-specific BCA activation, reducing unnecessary microbial dispersal and enhancing disease suppression [225]. These next-generation nano-formulations could provide more effective protection against postharvest pathogens while minimizing waste and application frequency [226]. Additionally, the use of bioengineered nano-materials derived from plant-based polymers or microbial exopolysaccharides can improve the biocompatibility of Nano-BCAs, ensuring their long-term viability in agricultural systems without adverse environmental impacts [227,228].

Future research should also focus on integrating Nano-BCAs with precision agriculture and digital farming technologies to enhance their application efficiency and real-time monitoring capabilities [229]. The use of nanobiosensors for pathogen detection could enable early diagnosis of postharvest infections, facilitating targeted application of Nano-BCAs at critical intervention points [187]. By coupling nanotechnology with artificial intelligence (AI) and machine learning, it may be possible to predict disease outbreaks, optimize BCA deployment, and automate postharvest disease management strategies [230]. Remote sensing technologies and nano-enabled spray systems can further enhance the precision of Nano-BCA applications, reducing waste while ensuring uniform coverage on fruit and vegetable surfaces [227]. The development of electrostatic or magnetically guided nano-formulations may also improve the adhesion of BCAs to produce surfaces, preventing wash-off and enhancing long-term protection against pathogens [231].

Another critical area of future research involves ensuring the safety, regulatory compliance, and consumer acceptance of Nano-BCAs [232]. Although biodegradable nanocarriers such as chitosan, starch-based nanoparticles, and lipid-based vesicles have demonstrated low toxicity and environmental compatibility, the long-term fate and persistence of nanoparticles in food systems require further investigation [188]. Regulatory agencies such as the USFDA and the EFSA require comprehensive risk assessments for nanotechnology-based formulations before their commercial adoption [233]. Developing standardized safety protocols, harmonized regulatory guidelines, and internationally accepted nano-toxicity assessment methods will be essential for ensuring the widespread approval and commercialization of Nano-BCAs [234]. Additionally, public perception of nanotechnology in food systems remains a key determinant of market success, necessitating transparent risk communication, consumer education, and clear labeling policies to build trust in Nano-BCA applications [216].

To achieve large-scale adoption, future studies must focus on cost-effective and scalable manufacturing processes for Nano-BCAs [154]. While nanotechnology has proven effective in enhancing BCA viability, high production costs remain a barrier to widespread implementation in agriculture [161]. The use of agro-industrial by-products as raw materials for nanocarrier synthesis can help lower costs while promoting circular economy practices [202]. Optimizing nanoencapsulation methods, improving fermentation-based nanoparticle production, and developing low-energy processing techniques will also contribute to making Nano-BCAs more economically viable [235]. Additionally, partnerships between academic researchers, industry stakeholders, and agricultural policy-makers will be crucial in fostering technology transfer and creating market-driven solutions for sustainable Nano-BCA deployment [236].

The future of Nano-BCAs in sustainable agriculture will also depend on their ability to support global food security and climate resilience [187]. The continued rise in postharvest losses due to climate change-related stressors, such as increased temperature variability and humidity fluctuations, underscores the need for adaptive biocontrol solutions [49]. Nano-enhanced BCAs with multi-functional properties, such as improved stress tolerance, enhanced biofilm formation, and synergistic antimicrobial effects, could provide more resilient and adaptable solutions for pathogen control in diverse agro-climatic conditions [20]. Additionally, incorporating Nano-BCAs into IPM programs alongside biological pesticides, biofungicides, and plant-derived antimicrobials will create holistic, multi-layered defense systems against postharvest spoilage pathogens [20].

Addressing existing limitations and harnessing emerging technological advancements, Nano-BCAs can play a transformative role in reducing global food losses, improving supply chain efficiency, and promoting eco-friendly agricultural practices. The transition from conventional chemical-based disease management toward nanotechnology-driven biocontrol solutions will require a multidisciplinary approach, bringing together expertise from nanotechnology, microbiology, food safety, agricultural engineering, and regulatory sciences [237,238]. As innovation continues, the successful integration of Nano-BCAs into sustainable agriculture will pave the way for safer, more efficient, and environmentally responsible food production systems, ensuring long-term benefits for producers, consumers, and the global ecosystem [239,240,241].

## 10. Conclusions

Postharvest diseases continue to pose a significant threat to global fruit and vegetable supply chains, leading to extensive losses in quality and quantity. Biological control agents, including beneficial bacteria, fungi, and yeasts, have demonstrated considerable potential in mitigating postharvest pathogens through mechanisms such as antibiosis, competition, mycoparasitism, and induction of host resistance. Their environmentally friendly nature and minimal impact on food safety make them attractive alternatives to chemical fungicides. However, the effectiveness of BCAs can be limited by environmental variability, formulation stability, and delivery challenges, which restrict their large-scale commercial application.

To overcome these limitations, nanotechnology offers innovative enhancements that improve the functionality and performance of BCAs. Nanoformulations can provide controlled release, improved shelf-life, targeted delivery, and protection from environmental stress, thereby amplifying the antimicrobial efficacy of BCAs. Techniques such as nanoencapsulation and nanocomposite films not only protect microbial viability but also enable site-specific interactions with postharvest pathogens. The synergistic integration of BCAs and nanotechnology, referred to as Nano-BCAs, represents a promising frontier for developing sustainable, efficient, and scalable postharvest management systems. Future research should focus on risk assessments, regulatory frameworks, and cost-effective manufacturing to facilitate the safe deployment of these advanced biocontrol strategies in global food systems.

## Data Availability

No new data were created or analyzed in this study. Data sharing is not applicable to this article.
